# Optimization for Software Implementation of Fractional Calculus Numerical Methods in an Embedded System

**DOI:** 10.3390/e22050566

**Published:** 2020-05-18

**Authors:** Mariusz Matusiak

**Affiliations:** Institute of Applied Computer Science, Lodz University of Technology, 90-924 Lodz, Poland; mmatusiak@iis.p.lodz.pl

**Keywords:** fractional-order differential equations, Grünwald–Letnikov differintegral, fractional-order backward difference/sum, Arm^®^ Cortex^®^-M, STM32^TM^ microcontroller implementation

## Abstract

In this article, some practical software optimization methods for implementations of fractional order backward difference, sum, and differintegral operator based on Grünwald–Letnikov definition are presented. These numerical algorithms are of great interest in the context of the evaluation of fractional-order differential equations in embedded systems, due to their more convenient form compared to Caputo and Riemann–Liouville definitions or Laplace transforms, based on the discrete convolution operation. A well-known difficulty relates to the non-locality of the operator, implying continually increasing numbers of processed samples, which may reach the limits of available memory or lead to exceeding the desired computation time. In the study presented here, several promising software optimization techniques were analyzed and tested in the evaluation of the variable fractional-order backward difference and derivative on two different Arm^®^ Cortex^®^-M architectures. Reductions in computation times of up to 75% and 87% were achieved compared to the initial implementation, depending on the type of Arm^®^ core.

## 1. Introduction

Fractional calculus is an increasingly valuable and efficient mathematical tool used in various fields of science and engineering for the synthesis of highly accurate models of real dynamic systems, developing precise control algorithms, and performing complex signal processing [1,2,3]. More and more novel applications can be found nowadays in all fields of science and engineering. Apart from classic examples like the modeling of viscoelastic materials [4] and the diffusion process [5,6], it was also found to be useful in economics for modeling of financial systems and economic growth [7,8], in medicine and biomedical engineering for disease analysis, drug modeling and administration, and signal acquisition [9,10,11,12,13], in computer science [14] for the development of neural networks with memory effects, and other applications with time-varying values of fractional-orders [15]. Only in the field of electrical engineering do some recent advances involve mathematical descriptions of supercapacitors [16,17], lithium-ion batteries with nonlinear capacities [18], and nonlinear coils in a ferroresonant circuit [19]. In control engineering, the algorithms of fractional-order controllers have received much interest, in particular, the fractional-order PID controller [20,21,22]. Due to the availability of a higher number of degrees of freedom (additional two), the more robust control may, in principle, be applied, and desired plant response characteristics thereby achieved more rapidly [23]. The difficulty of this process, however, is twofold: firstly, the digital implementation of the fractional-order system, associated with the problem of continuously, linearly growing numbers of calculations; secondly, the proper selection of values for fractional differentiation and integration orders. Finite memory available in a target embedded system and the fixed sampling frequency in real-time applications require limiting the operations, making the implementation less accurate and more error-prone. In order to resolve these issues, one should consider optimizing the fractional-calculus-based algorithms, which is usually not essential for simulations performed in computational software such as MATLAB. For the Grünwald–Letnikov definitions of backward difference/sum and differintegral, a widely applied approach is the Short Memory Principle, introduced by Podlubny in [3]. Under specific conditions, however (selected fractional order, range and frequency of the input signal, small buffer sizes), truncation of past samples may produce significant numerical errors [24,25]. Therefore, some additional optimization is required. This question is particularly crucial when even more complex algorithms, based on fractional calculus, are considered. One of the examples may be a closed-loop control system with a variable fractional-order PID controller with adapting differentiation and integration orders.There are several types of remedies to this issue.

The first type is a group of various Short Memory methods. The equivalent form of the Grünwald–Letnikov differintegral, based on the Horner scheme, was demonstrated, e.g., in [21]. In [26], a simplification was proposed based on dividing a series of coefficients into *l* parts and substituting groups of them with constant values. In [6], the adaptive time steps memory method was introduced, whereby samples in the past are taken at consecutively longer intervals with higher weights.

The second type involves approximation in the frequency domain of the Laplace fractional-order operator $G\left(s\right)=s^{\nu }$. Commonly applied techniques are Oustaloup Recursive filter (ORA) [27,28,29] and continued fraction expansion (CFE) [30,31], with later reduction of the polynomial order using the balancing reduction technique [32,33].

In the present article, the focus lies on an additional level of optimization, which is software optimization, universal from the perspective of any selected fractional-order numerical algorithm. Depending on the selected target architecture, several different programming techniques for improving algorithm efficiency may be implemented. The considered solutions imply the use of Arm^®^ CMSIS-DSP library with *intrinsic* and Single Instruction Multiple Data (SIMD) functions, as well as other hardware extensions. The research aimed to obtain the highest possible performance, while preserving the ease of middle-level C programming, ensuring software portability and omitting CPU-specific assembly code snippets. Several iterations of the tests were conducted using two 32-bit RISC Arm^®^ Cortex^®^-M microcontrollers manufactured by STMicroelectronics. First, the implementations of fractional-order backward difference and derivative using constant single-precision floating-point $a_{j}^{\nu }$ binomial coefficients for varying buffer sizes of $L_{1}=32$ and $L_{2}=256$ values were analyzed. The memory limitations of the microcontrollers were also investigated. Next, the performance of initial algorithms for fractional backward difference/sum and differentiator/integrator of variable orders was measured. The latter is particularly useful for the realization of adaptive fractional-order ${\mathrm{PI}}^{\mu (t)}$$D^{\nu (t)}$ controllers with variable orders of I and D terms. The algorithms were then optimized using the described techniques. In the final step, fixed-point arithmetic with the conversion of numbers to Qm.n notation [34] with m bits for the integer part and n bits for the fractional part was applied.

The paper is organized as follows. Section 2 sets out mathematical preliminaries, including the Grünwald–Letnikov definitions of fractional-order backward difference/sum and differintegral. In Section 3, the characteristics of the hardware testing platform are presented. Section 4 provides a detailed description of the algorithms and their implementation. In Section 5, optimization techniques are proposed, and the results of the conducted experiments are presented. In Section 6, the alternative approach using fixed-point arithmetic is discussed. Related software is published as the Supplementary Materials to this paper.

## 2. Mathematical Preliminaries

**Definition** **1.** *Grünwald–Letnikov fractional-order backward difference/sum (GL-FOBD/S).*

*Let $n,j,k\in N_{0}$, $N_{0}=N\cup \left\{0\right\}$. Classic n-th order backward difference of a function f(t), defined in discrete time instants t=kh is given by the formula:*

(1) ∇knft=∑j=0n−1jnjf((k−j)h)

*where nj is a binomial coefficient and $h\in R_{+}$ is a finite, constant sampling period. Replacing the order n with an arbitrary real one $\nu \in R_{+}$ results in a definition of the Grünwald–Letnikov fractional-order backward difference [1,3]:*

(2)  GLΔkνf(t)=∑j=0∞(−1)jνjf((k−j)h)=∑j=0∞(−1)j(ν(ν−1)…(ν−j+1))j!f((k−j)h)==∑j=0∞(−1)jΓ(ν+1)j!Γ(ν−j+1)f((k−j)h)=∑j=0∞ajνf((k−j)h)

*where *Γ* is the Euler’s gamma function and $a_{j}^{\nu }$ denotes discrete coefficients also known as the oblivion function [35]. In practice, the function f(t) is defined for $t\in [t_{0},+\infty )$, where $t_{0}$ is considered as a starting point. Hence, the infinite sum in Equation (2) is limited by $N=\left\lceil \frac{\left(t-t_{0}\right)}{h}\right\rceil$. The recursive formula for $a_{j}^{\nu }$ is defined as:*

(3) ajν=(−1)jνj=1forj=0aj−1ν(1−1+νj)forj=1,2,…

*Fractional-order backward sum is obtained for negative values of the order $\mu \in R_{-}$, e.g., μ=−ν*

**Definition** **2.** *Grünwald–Letnikov variable fractional-order backward difference/sum (GL-VFOBD/S).*

*Let the fractional order value be represented as a discrete function ν(t). The function replaces the constant value ν in Equations (1)–(3). This generalization is known as the variable fractional-order backward difference/sum. Using the time-varying oblivion function $a_{j}^{\left[\nu (t)\right]}$, one may calculate in each step its value for f(t) [36] with:*

(4) $$\overset{GL}{t_{0}\;}\Delta_{t}^{\left[\nu (t)\right]}f\left(t\right)=\sum_{j=0}^{N}a_{j}^{\left[\nu (t)\right]}f\left(\left(k-j\right)h\right)$$

**Definition** **3.** *Grünwald–Letnikov variable fractional-order derivative/integral (GL-VFOD/I).*

*On the basis of GL-VFOBD/S the differintegral operator is defined by Equation (5):*

(5) $$\overset{GL}{t_{0}\;}D_{t}^{\left[\nu (t)\right]}f\left(t\right)=\lim_{h\to 0^{+}}\frac{\overset{GL}{t_{0}\;}\Delta_{t}^{\left[\nu (t)\right]}f\left(t\right)}{h^{\left[\nu (t)\right]}}$$

*For microcontroller implementations, based on the assumption that h is as small as possible, this may be approximated to:*

(6) $$\overset{GL}{t_{0}\;}D_{t}^{\left[\nu (t)\right]}f\left(t\right)\approx \frac{\overset{GL}{t_{0}\;}\Delta_{t}^{\left[\nu (t)\right]}f\left(t\right)}{h^{\left[\nu (t)\right]}}$$

Non-local fractional operator leads to the increasing number of calculations required to evaluate the result each step. In the *k*-th step, kM instructions have to be performed by a microcontroller, where *M* denotes the number of instructions for processing a single sample. At a certain step, either the time required to complete the calculations exceeds the applied constant sampling period *h* ($t_{kM}>h$), or the limit of the memory allocated for the buffers is reached. One of the Short Memory solutions is to limit the number of samples to *L*, so the time of calculations $t_{LM}$ is always shorter than *h*. Thus, the oblivion function is amended to [26]:

(7) $$\begin{array}{c} a_{j}^{\nu }=\left\{\begin{array}{cc} 1 & \mathrm{for}\;j=0\\ {\left(-1\right)}^{j}\frac{\left(\nu (\nu -1)\ldots (\nu -j+1)\right)}{j!} & \mathrm{for}\;0<j\leq L\\ {\left(-1\right)}^{L}\frac{\left(\nu (\nu -1)\ldots (\nu -j+1)\right)}{L!} & \mathrm{for}\;j>L \end{array}\right. \end{array}$$

Accuracy of the second solution depends on the selected value of fractional-order and may be applied when the coefficients drop rapidly to zero (ν>0). Then, one can approximate the result by limiting the number of multiplications to *L*:

(8) $$\begin{array}{c} a_{j}^{\nu }=\left\{\begin{array}{cc} 1 & \mathrm{for}\;j=0\\ {\left(-1\right)}^{j}\frac{\left(\nu (\nu -1)\ldots (\nu -j+1)\right)}{j!} & \mathrm{for}\;0<j\leq L\\ 0 & \mathrm{for}\;j>L \end{array}\right. \end{array}$$

## 3. Description of the Hardware Testing Platform

Tests were conducted using two STM32^TM^ microcontrollers, models: STM32L152RCT6 [37] and STM32F746ZG [38], designed on the basis of popular 32-bit cores: Arm^®^ Cortex^®^-M3 and Cortex^®^-M7, respectively. The main differences between these devices lie in the availability of a hardware floating-point unit (FPU) and a higher maximum CPU clock frequency in the case of the STM32F746ZG. The microcontrollers are distributed in STM32^TM^ Discovery and Nucleo-144 kits [39,40]. Their key features are listed in Table 1.

The motivation for selecting these models was to test the performance of the evaluation of fractional-order differential equations using platforms widely applied in industrial applications, equivalent to expensive DSP processors. Cortex^®^-M microcontrollers usually do not reach the same computation power, often due to much lower CPU clock frequency (e.g., the TMS320C6678 DSP processor operates at 1.4 GHz). However, they have been equipped with numerous extensions for accelerating calculations, including Single Instruction, Multiple Data (SIMD) operations, optimized multiply-accumulate (MAC) and DSP instructions, direct memory access (DMA), and hardware floating-point units (FPU). A significant advantage is the availability of basic peripherals, memories, communication interfaces, and power regulators. This offers a low-cost alternative to multi-core systems, with each core dedicated to specific tasks (e.g., primary DSP core to signal processing tasks and secondary core to an operating system, communication with external peripherals and power management).

The STM32F746ZG MCU belongs to the High-Performance STM32F7 series. The efficiency of the Cortex^®^-M7 core has been increased by a 6-stage dual-issue pipeline capable of processing two instructions per clock cycle. At the fourth stage of the pipeline (*Issue*), processed instructions are split and further executed by one of the separated dedicated blocks—an arithmetic logic unit with a SIMD extension, MAC pipeline, single-precision floating-point pipeline, or branch prediction block.

The STM32L152RCT6 was designed using STM32^TM^ ultra-low-power technology. The primary feature of the microcontroller is the availability of several low power modes dedicated to battery-powered applications (in Standby mode current consumption is reduced to only 0.29 μA). Maximum performance is therefore limited to only 33DMIPS at a clock frequency of 32 MHz. Due to the lack of hardware floating-point unit, all operations on real numbers are software emulated, which strongly affects the computation time.

## 4. Implementation of the Grünwald–Letnikov Fractional-Order Operator

### 4.1. Memory Limitations

Before implementing numerical methods for evaluation of the Grünwald–Letnikov operator, the maximum reference size for memory allocated to the f(kh) samples and $a_{j}^{\nu }$ coefficients of a single-precision type *float* was determined for STM32L152RCT6 microcontroller. A test program for recursive calculation of $a_{j}^{\nu }$ coefficients using Equation (3) was written in C language and executed for various buffer lengths *L*. In addition to the memory reserved for buffers, at each *k* step, more SRAM memory was also consumed by the stack in recursive function calls. The maximum measured length that did not lead to a hard fault error caused by memory access violation was 454. Thus, further tests were performed for $L_{1}=32$ and $L_{2}=256$ buffer lengths.

### 4.2. Compiler Settings

Programs were written in System Workbench for STM32 IDE v2.9.1 and compiled using the Arm^®^ Embedded GCC compiler, distributed as part of the GNU Arm^®^ Embedded Toolchain v9.2. Several available optimization levels were tested, starting with the default –O0 (no optimizations) flag. In that mode, instructions are translated by the compiler line by line, and breakpoints can be placed and hit anywhere in the executable code. This level is most suitable for the software development process, providing the most accurate debugging experience and the possibility of reading and modifying variables at a debug session. The second level was –O2, which is the highest standard-compliant optimization level that does not introduce a trade-off between the size of the program and its execution speed. This option is commonly enabled in the release building profiles of numerous GNU projects, including the Linux kernel. The last level tested was –O3, in which more code optimizations are applied, however, usually at the cost of the increased size of the output binary. This is an outcome of functions inlining and loops unrolling. Therefore, the program may not become faster in all cases. The Arm^®^ GCC also supports the more aggressive –Ofast optimization level, which replaces math operations with their fast modifications. However, due to the generation of non-standard-compliant code and potential software vulnerabilities, this setting was not taken into consideration. A detailed description of all compiler optimization options can be found in the GCC user manual [41].

### 4.3. Measuring the Performance

The family of Arm^®^ Cortex^®^ microcontrollers is equipped with a peripheral called the Data Watchpoint and Trace (DWT) unit [42]. It contains up to six different counters and four hardware comparators, which can serve as a source for event triggering (Embedded Trace Macrocell, PC sampler, and data address sampler triggers) and configuring hardware watchpoints. Counting the number of elapsed core cycles is also possible and done by reading the values stored in the DWT Cycle Count (DWT_CYCCNT) register. Combining it with the value of CPU clock frequency $F_{CPU}$, one can determine the time of a specific set of operations as $t_{op}=\frac{c_{2}-c_{1}}{F_{CPU}}$, where $c_{1},c_{2}$ denote the number of cycles read from the DWT_CYCCNT register before and after the analyzed section, respectively. The CYCCNT counter is counting upwards to $2^{32}$ and wraps around to 0. The configuration procedure for Data Watchpoint and Trace unit is as follows:

1. TRCENA bit [24] in the Debug Exception and Monitor Control Register (DEMCR) set to 1 to enable use of the trace and debug blocks.
2. CYCCNTENA bit [0] in the DWT Control Register (DWT_CTRL) set to 1 to enable the CYCCNT counter.
3. Value of the DWT_CYCCNT register initialized to 0.

The addresses of the registers may differ based upon the selected microcontroller model so verification in the relevant user manual is recommended.

### 4.4. Implementation of Fractional-Order Backward Difference

**Figure 1:**
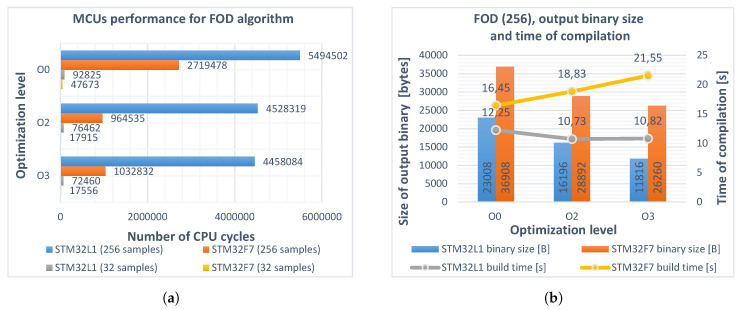
(**a**) performance of STM32L152RCT6 (blue, gray) and STM32F746ZG (red, yellow) microcontrollers as the number of executed CPU cycles, realizing the fractional-order differintegral (Equation (6)) (*ν_const_*(*t*) = 0.7) for different optimization levels O0, O2, O3, and buffer lengths *L*_1_, *L*_2_. Obtained improvement for both microcontrollers (worst case vs best case, buffer length *L*_1_): 22% and 63%, respectively; (**b**) sizes of the output binaries (columns) and compilation times (polylines) for different optimization levels of the program. Buffer length *L*_2_ = 256.

The definitions of the Grünwald–Letnikov backward difference described by Equations (2), (4), and derivative by Equation (6) were implemented in C language test programs for both microcontrollers. The code was initially compiled for reference without any optimization, using the -O0 parameter. Single-precision *float*-type $a_{j}^{\nu }$ coefficients were calculated for a fixed order value ν=0.7 at the beginning of the program, along with the required peripherals initialization routines. Example input signals representing a discrete unit step and a linear function were also generated. Calculation of the fractional-order backward difference using the $a_{j}^{\nu }$ coefficients and the response of fractional-order differentiator for sampling time h=0.5 s was executed in the main program loop. The maximum number of CPU cycles for each of the algorithms was measured every step for $L_{1}$ and $L_{2}$ buffer lengths separately. The procedure was repeated using -O2 and -O3 optimization flags. The results are presented in Figure 1a. The sizes of the output binaries as well as compilation times were also measured and are shown in Figure 1b. The process was performed on Dell Inspiron 5379 PC with Intel^®^ Core^TM^ i5-8250U, 16 GB of RAM and MS Windows^®^ 10 Pro operating system.

**Figure 2:**
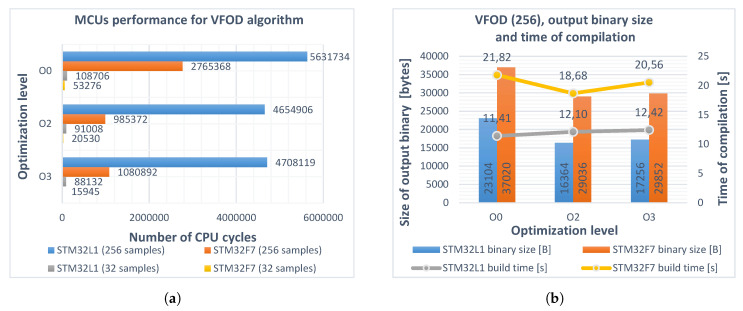
(**a**) performance of STM32L152RCT6 (blue, gray) and STM32F746ZG (red, yellow) microcontrollers realizing the variable fractional-order differintegral (Equation (6)) for different optimization levels O0, O2, O3 and buffer lengths *L*_1_, *L*_2_. Obtained improvement for both microcontrollers (buffer length *L*_1_): 19% and 70%, respectively; (**b**) sizes of the output binaries (columns) and compilation times (polylines) for different optimization levels of the program. Buffer length *L*_2_ = 256.

In the next attempt, a variable fractional order ν(t) was introduced. The $a_{j}^{\left[\nu (t)\right]}$ coefficients were recalculated before the backward difference and differentiator responses in the main program loop. The value of order ν was linearly incremented each step by small value $\Delta =+10^{-5}$. The results are presented in Figure 2.

The numbers of cycles and sizes of the output binaries in the second case were slightly increased due to the additional evaluation of $a_{j}^{\left[\nu (t)\right]}$. The impact of software emulated floating-point calculations in the STM32L152RCT6 microcontroller is clearly noticeable. Flags –O2 and –O3 in all cases reduced the computation time and program size, although in the case of the STM32F746ZG program processing 256 samples, the –O2 level gave better results than –O3. No significant differences in compilation times were observed. The results from the second attempt served as a reference for further optimizations.

## 5. Optimization

### 5.1. SIMD and DSP Instructions in the CMSIS Library

Analysis of the various discrete signal processing algorithms, including finite and infinite impulse response filtering, discrete Fourier transformation, correlation, and the studied fractional-order operators, clearly indicates that they are dominated by multiply-accumulate operations (MAC). In numerous architectures, MAC are optimized to take only a single CPU cycle. In the Cortex^®^-M architecture, this operation can be realized on 64- and 32-bit operands (64b←64b+32b×32b). Since the release of Cortex^®^-M4 core, Single Instruction Multiple Data (SIMD) extensions have also been supported. With SIMD, one can increase the processing capability by performing calculations simultaneously on multiple 4 × 8-bit or 2 × 16-bit operands. A complete list of the Cortex^®^ extensions can be found in [43].

SIMD, dedicated floating-point and digital signal processing (DSP) instructions, are among the features implemented in CMSIS-DSP library, distributed by Arm^®^ [44]. It contains over 60 different methods, optimized for various cores, endianness, and data types. Fixed-point (*Q7, Q15, Q31*) as well as single- and double-precision floating-point arithmetics (*float32_t*, *float64_t*) are supported.

In order to use CMSIS-DSP, the *<arm_math.h>* header file should be included and the proper precompiled *.lib file linked. For Cortex^®^-M4 and M7 microcontrollers, an explicit definition of the *__FPU_PRESENT* macro is required in order to enable support of the hardware FPU instructions.

The performance of three basic DSP algorithms using CMSIS-DSP on STM32F429 and STM32F746 microcontrollers has been measured and demonstrated in [45].

### 5.2. Enabling the Hardware Floating-Point Unit

For optimized implementation, it must be ensured that the floating-point unit available on the microcontroller has been enabled and properly initialized. Otherwise, without an explicit setup, the floating-point arithmetic may be software emulated, as is the default in the case of STM32F746ZG for reducing the power consumption [46]. The procedure is performed by setting the CP10 and CP11 coprocessor bits in the Coprocessor Access Control (CPACR) register (software), mode of the Application Binary Interface (ABI) to *hard* and version of the embedded FPU architecture to *fpv5-sp-d16* (options of the Arm^®^ GCC compiler). The ABI interface determines the type of registers which are used to pass real variables to the linked functions. The flag -mfloat-abi=*hard* corresponds to dedicated floating-point registers, while -mfloat-abi=*soft* (cross-platform compatible) to integer registers. The version of architecture can be specified either by –mfpu=*fpv5-sp-d16* for single-precision support or *fpv5-dp-d16* for double-precision support.

### 5.3. Other Optimizations

To improve performance further, other known techniques may be applied, including Lookup Tables (LUT), circular buffers, and static inline functions. If the value of fractional order is constant or equal to one of several predefined values and only part of the Flash memory is occupied, then $a_{j}^{\nu }$ coefficients can be precomputed and stored in the Flash. Static inline functions are internally linked by a compiler, and their code is substituted into caller functions. This technique might be unsafe in some implementations, causing a rapid increase in the program size e.g., when the function is called in a loop.

### 5.4. Implementation

The guidelines described above were used to optimize the implementation of computing $a_{j}^{\left[\nu (t)\right]}$ coefficients, variable fractional-order backward difference, sum, and derivative. Tests were performed with the same set of parameters, as described in Section 4. The following modifications were applied:

1. The appropriate linked CMSIS-DSP lib file: *arm_cortexM3l_math.lib* for STM32L152RCT6 (little-endian) and *arm_cortexM7lfsp_math.lib* for STM32F746ZG (little-endian, single-precision FPU). Required macros defined.
2. The implementation of convolution from Equation (4) from the previous step was replaced by the CMSIS *arm_conv_part_f32* function. In addition, the multiplication by $\frac{1}{h^{\left[\nu (t)\right]}}$ factor in Equation (6) was performed using the *arm_scale_f32* function.

**Figure 3:**
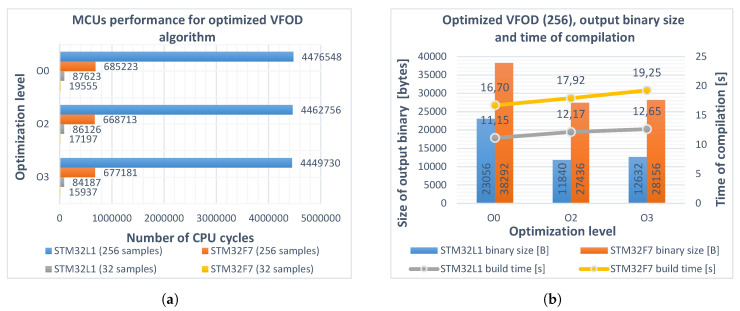
(**a**) performance of STM32L152RCT6 (blue, gray) and STM32F746ZG (red, yellow) microcontrollers realizing the modified implementation of variable fractional-order differintegral (Equation (6)) for different optimization levels O0, O2, O3 and buffer lengths *L*_1_, *L*_2_. Obtained improvement for both microcontrollers (buffer length *L*_1_): 4% and 19%, respectively; (**b**) sizes of the output binaries (columns) and compilation times (polylines) for different optimization levels of the program. Buffer length *L*_2_ = 256.

Significant increases in performance were observed for the Cortex^®^-M7. The number of CPU cycles was reduced by over 75% for the buffer length $L_{2}$ and for the program compiled with the same –O0 flag. The –O2 and –O3 levels generated even better machine code. For Cortex^®^-M3, the improvement was smaller but also noticeable, resulting in a reduction of the execution time up to 19% and 20% for the buffers with 32 and 256 samples, respectively. Details are presented in Figure 3.

## 6. Fixed-Point Arithmetic

In the fixed-point arithmetic, the Qm.n notation proposed by Texas Instruments^TM^ [34] is used to represent real numbers devoting a constant number of *m* bits for integer parts and a constant number of *n* bits for fractional parts. One additional bit is reserved for a sign, and a position of the radix point is fixed. Numbers are stored in integer registers, and all calculations are performed using standard hardware arithmetic logic unit. The range of a Qm.n number is defined as $\left[-2^{m-1},2^{m-1}-2^{-n}\right]$ and the resolution equals $2^{-n}$. Fixed-point has been used most often for low-cost or older microcontrollers without hardware floating-point units but is also implemented in many high-end DSP applications to increase the overall performance of the software. The drawbacks of this approach include potential issues with saturation, precision loss, or selecting an insufficient range of numbers. Thus, more complex implementation capable of handling normalization (bit-shifting) and bounds checking is required. Additional scaling of real numbers may also be needed.

To compare the performance of the fixed-point arithmetic with the previously described floating-point approach, the following assumptions were made: in order to avoid saturation, the 32-bit signed integer *q31_t* type was used for storing numbers in *Q11.21* format, giving a range of [−1024, 1024–2^−21^] and a resolution of $2^{-21}\left(4.768e-7\right)$. This allowed Equation (2) to be implemented without scaling the maximum number of coefficients (256) and provided a satisfactory resolution. It needs to be stressed, however, that, for different applications, the above requirements will have to be adapted. As the CMSIS-DSP library supports only *Q1.31*, *Q1.15*, and *Q1.7* formats, the author’s implementation of the fixed-point arithmetic was introduced. The procedure was as follows:

1. The vector of the predefined floating-point input samples, initial fractional-order $\nu_{0}=0.7$, and the sampling time *h* were converted to *Q11.21* format by multiplying the values by $2^{21}$ and rounding to the nearest integer.
2. The recursive function for calculating $a_{j}^{\left[\nu (t)\right]}$ and fractional differintegral algorithm were modified for handling fixed-point arithmetic in *Q11.21* notation.
3. In the main loop, the $\nu_{Q11.21}$ order was incremented by one each step and the vectors of the $a_{j_{Q11.21}}^{\left[\nu (t)\right]}$ coefficients, as well as the variable fractional-order backward difference and derivative responses, were recalculated.

**Figure 4:**
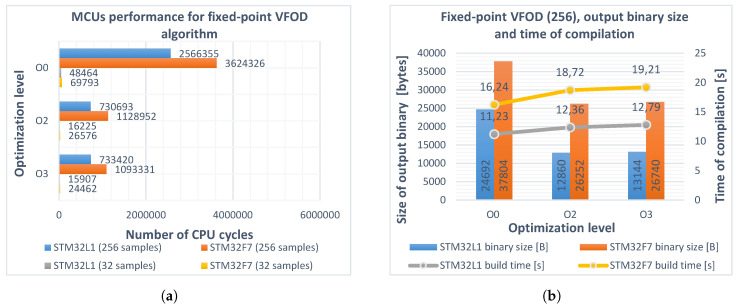
(**a**) performance of STM32L152RCT6 (blue, gray) and STM32F746ZG (red, yellow) microcontrollers realizing the fixed-point implementation of variable fractional-order differintegral (Equation (6)) for different optimization levels O0, O2, O3 and buffer lengths *L*_1_, *L*_2_. Obtained improvement for both microcontrollers (buffer length *L*_1_): 67% and 65%, respectively; (**b**) sizes of the output binaries (columns) and compilation times (polylines) for different optimization levels of the program. Buffer length *L*_2_ = 256.

Results were later converted back to *float* type and verified using the open-source Kibo toolbox [47]. This time a significant increase in efficiency can be observed in the Cortex^®^-M3 case (see Figure 4). Calculation time was reduced by over 84% for both sizes of the buffers with –O2 or –O3 optimization levels applied. Moreover, Cortex^®^-M3 was found to be faster (assuming the same CPU clock frequency) than Cortex^®^-M7 at executing the same algorithm.

## 7. Conclusions

In this paper, several approaches have been presented for optimizing microcontroller implementations of variable fractional-order backward difference, sum, and differintegral. A notable improvement was achieved for the STM32F746ZG microcontroller by using the CMSIS-DSP library, SIMD extensions, and a hardware floating-point unit. The performance of the STM32L152RCT6 was reduced, mostly by the software emulated floating-point arithmetic; however, the conversion to the fixed-point *Q11.21* improved it significantly. It should be noted that the fixed-point arithmetic involves more complex implementation, verification of the results, and requires additional conversion of variables when used in real-time applications with ADC and DAC converters. The size of the program and the execution speed were improved further by the proper configuration of the compiler optimization options. For the tested implementations, the –O2 level provided the best results in most cases. By analyzing the worst-case and the best-case scenarios, a conclusion can be drawn that in case of STM32L152RCT6 the best combination was the fixed-point implementation compiled with –O2 flag (87% reduction of the computation time), whereas, for STM32F746ZG, the CMSIS and hardware FPU-based implementation, also compiled with –O2 (75% reduction). Like mentioned in Section 4.2, the –O3 level in many cases generated larger code resulting in longer execution times. With different algorithms and requirements, other levels should be tested. The presented results may serve as a good starting point for further research on the implementation of more complex fractional-calculus-based algorithms in embedded systems, including fractional-order PID control with orders varying in time (VFOPID). In future work, optimizations of the numerical methods will be investigated, including the parallel implementation on multicore architectures for applications in closed-loop control systems.

## Figures and Tables

**Table 1:** Parameters of the selected microcontrollers.

Parameter Name	STM32L152RCT6 (Arm^®^ Cortex^®^-M3)	STM32F746ZG (Arm^®^ Cortex^®^-M7)
CPU clock frequency ($F_{CPU}$)	up to 32 MHz	up to 216 MHz
Memory (Flash,SRAM)	256 KB Flash + 32 KB SRAM + 8 KB EEPROM	1024 KB Flash + 320 KB SRAM
Converters (ADC,DAC)	12-bit 1 MSPS ADC, 12-bit DAC	3× 12-bit 2.4 MSPS ADC, 2× 12-bit DAC
Power supply ($V_{DD}$)	1.65–3.6 V	1.8–3.6 V
Other features	ultra-low-power technology, LCD driver, touch sensor channels	floating-point unit real-time accelerator, DSP instructions, LCD and cam interface

## Supplementary Materials

The following are available online at https://www.mdpi.com/1099-4300/22/5/566/s1.

## Abbreviations

The following abbreviations are used in this manuscript:

ABI	Application Binary Interface
CPACR	Coprocessor Access Control Register
DEMCR	Debug Exception and Monitor Control Register
DFT	Discrete Fourier Transform
DWT	Data Watchpoint and Trace unit
DWT_CTRL	DWT Control Register
DWT_CYCCNT	DWT Cycle Count Register
FIR	Finite Impulse Response
FPU	Floating-Point Unit
GL	Grünwald–Letnikov
IIR	Infinite Impulse Response
MAC	Multiply-Accumulate
PID	Proportional-Integral-Derivative Controller
SIMD	Single Instruction Multiple Data
(V)FOBD/S	(Variable) Fractional-Order Backward Difference/Sum
(V)FOD/I	(Variable) Fractional-Order Differintegral
(V)FOPID	(Variable) Fractional-Order Proportional-Integral-Derivative Controller
